# Data analysis on physical and mechanical properties of cassava pellets

**DOI:** 10.1016/j.dib.2017.11.044

**Published:** 2017-11-16

**Authors:** Pelumi E. Oguntunde, Oluyemisi A. Adejumo, Oluwole A. Odetunmibi, Hilary I. Okagbue, Adebowale O. Adejumo

**Affiliations:** a Department of Mathematics, Covenant University, Ota, Nigeria; b AIDE Department, National Centre for Agricultural Mechanization, Ilorin, Nigeria; c Department of Statistics, University of Ilorin, Ilorin, Nigeria

**Keywords:** Moisture content, Machine speed, Die diameter, Hardness, Durability, Bulk density, Unit density, Cassava pellets, Cassava dough

## Abstract

In this data article, laboratory experimental investigation results carried out at National Centre for Agricultural Mechanization (NCAM) on moisture content, machine speed, die diameter of the rig, and the outputs (hardness, durability, bulk density, and unit density of the pellets) at different levels of cassava pellets were observed. Analysis of variance using randomized complete block design with factorial was used to perform analysis for each of the outputs: hardness, durability, bulk density, and unit density of the pellets. A clear description on each of these outputs was considered separately using tables and figures. It was observed that for all the output with the exception of unit density, their main factor effects as well as two and three ways interactions is significant at 5% level. This means that the hardness, bulk density and durability of cassava pellets respectively depend on the moisture content of the cassava dough, the machine speed, the die diameter of the extrusion rig and the combinations of these factors in pairs as well as the three altogether. Higher machine speeds produced more quality pellets at lower die diameters while lower machine speed is recommended for higher die diameter. Also the unit density depends on die diameter and the three-way interaction only. Unit density of cassava pellets is neither affected by machine parameters nor moisture content of the cassava dough. Moisture content of cassava dough, speed of the machine and die diameter of the extrusion rig are significant factors to be considered in pelletizing cassava to produce pellets. Increase in moisture content of cassava dough increase the quality of cassava pellets.

**Specification Table**

**Table:** 

**Subject area**	*Engineering and Bio-system*
**More specific subject area**	*Post Harvest, Food Process, Biomass and Bioenergy*
**Type of data**	*Tables and figures*
**How data was acquired**	*Unprocessed secondary data*
**Data format**	*Laboratory experimental investigation results on moisture content, machine speed, die diameter of the rig, and the outputs (hardness, durability, bulk density, and unit density of the pellets)*
**Experimental factors**	*Moisture content, machine speed, die diameter of the rig*
**Experimental features**	*Computational analysis: Analysis of variance (ANOVA), Randomized Complete Block Design with Factorial Experiment, Histogram.*
**Data source location**	*Agro-Industrial Development and Extension (AIDE) Department, National Centre for Agricultural Mechanization (NCAM), Idofian, Ilorin, Nigeria.*
**Data accessibility**	*All the data are in this data article as a*Supplementary data file
**Software**	*SPSS Statistical program and Microsoft Excel*

**Value of the data**

• The data on cassava pellets is useful for the Agencies saddled with the statutory responsible of food Storage and preservation.
• The data can be useful for policy makers in area of food security. This is due to the high level of cassava consumption among the populace in sub-Sahara Africa.
• The data is a good indicator for entrepreneurs or market operators dealing in the exporting cassava inform of pellets.
• The data can be useful in post- harvest and bio-system engineering studies.
• The data will be useful in biomass and bioenergy researches especially in the area of biofuel.
• The data are for educational purposes and food processing assessment studies.
• The unit density in the data is a measure parameter.
• The data can be used to determine the durability of conversion of cassava dough into pellets.
• The data can be useful in processing poultry feeds into pellets form.
• Several known statistical models, for example, Complete Randomized Design (CRD), factor analysis design, multiple regression, can be applied which provides alternatives to Randomised Complete Block Design with factorial experiment.

## 1 Data

The data for this paper were obtained from AIDE Department, National Centre for Agricultural Mechanization (NCAM), Idofian, Ilorin, Nigeria. The data are on experimental investigation performed on Cassava. Three factors were involved, each with four levels: moisture content (48.5%(wb), 50.5%(wb), 52.5%(wb), 54.5%(wb)); machine speed (1.5 mm|min, 2.5 mm|min, 3.5 mm|min, 4.5 mm|min); die diameter of the rig (6 mm, 8 mm, 10 mm, 12 mm), and each combination of this experiment (4^3^) were replicated three times. Altogether there were 192 experimental units. The analysis was done using 4^3^ factorial design with randomized complete block design.

The raw data with the three factors: moisture content, speed, die diameter, and their replication, and also each of the four outputs: hardness, bulk density, durability and unit density. Altogether, there are eight (8) columns and 192 rows, in the file, which can be assessed as Supplementary data.

Statistical summary of each of the outputs: hardness, bulk density, durability, unit density are presented in Table 1. It was observed that the average hardness in N, bulk density in kg|m^3^; durability in % and unit density of cassava pellets are 10.9505, 56.9264, 31.4840 and 0.0120 respectively.

**Table 1:** Summary statistics of the hardness, bulk density, durability and unit density of cassava pellets.

Statistic		Hardness	Bulk density	Durability	Unit density
	N	192	192	192	192
				
	Missing	0	0	0	0
Mean		10.9505	56.9264	31.4840	0.0112
Median		10.0000	55.6800	31.3400	0.0082
Mode		5.0000	45.6400	27.5900	0.0055^a^
Std. Deviation		6.7222	6.8852	12.0979	0.0146
Variance		45.1870	47.4050	146.3600	0.0000
Skewness		1.3430	0.2210	0.0180	5.7720
Std. Error of Skewness		0.1750	0.1750	0.1750	0.1750
Kurtosis		2.0360	-0.6260	0.0480	39.3270
Std. Error of Kurtosis		0.3490	0.3490	0.3490	0.3490
Minimum		3.0000	45.6400	7.6900	0.0013
Maximum		40.0000	73.0200	61.6400	0.1400
					
Percentiles	25	5.0000	52.0300	25.0450	0.0059
	50	10.0000	55.6800	31.3400	0.0082
	75	15.0000	62.9800	38.4600	0.0141

a Multiple modes exist. The smallest value is shown.

**Fig. 1:**
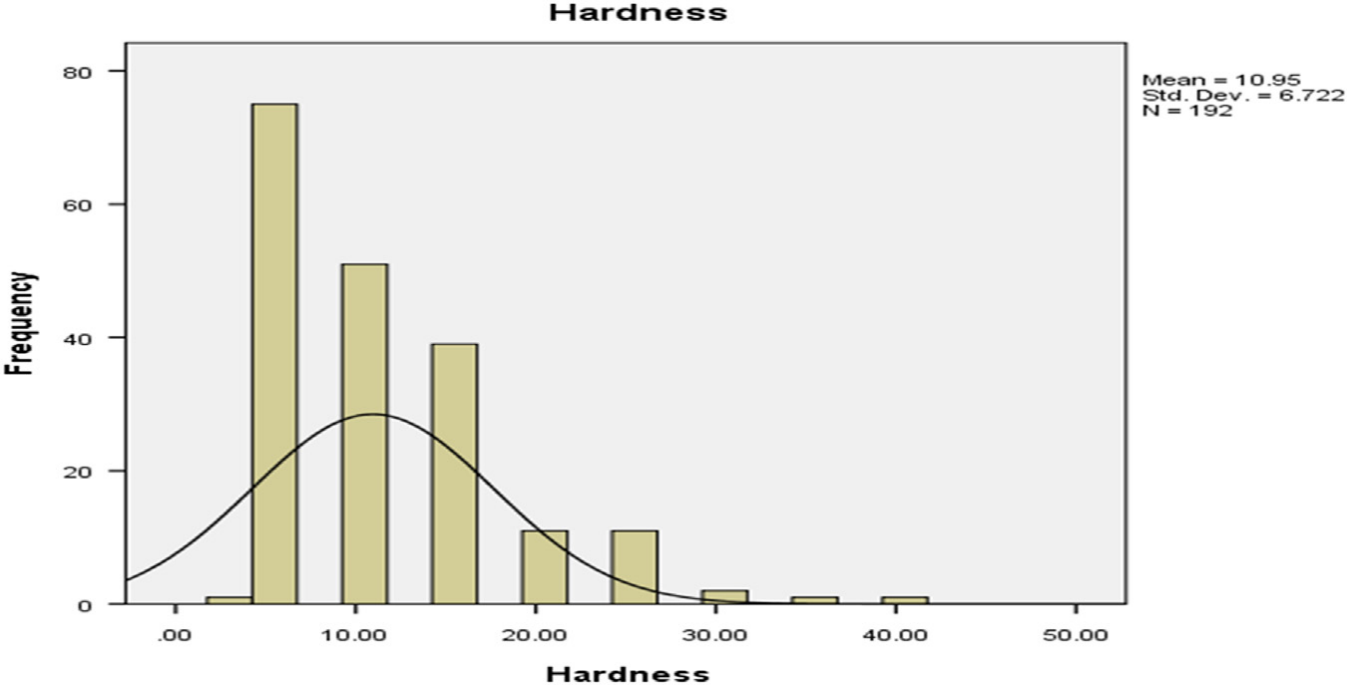
The hardness of cassava pellets.

**Fig. 2:**
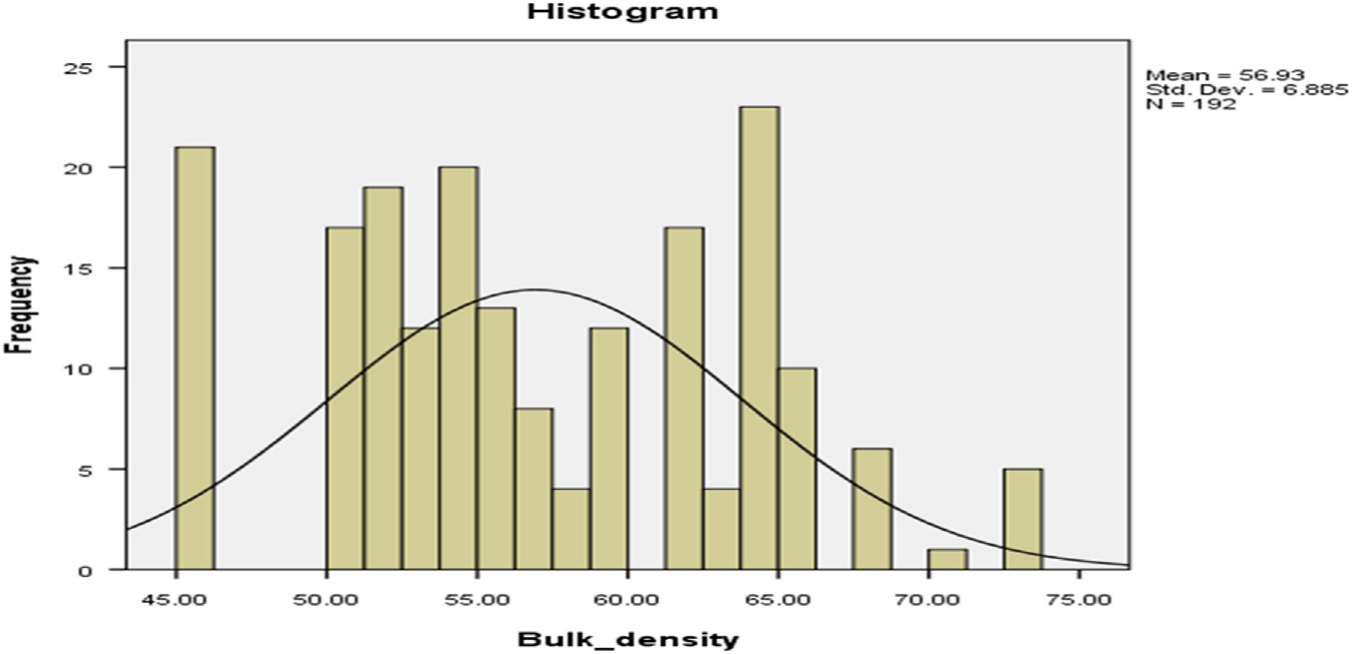
The bulk density of cassava pellets.

**Fig. 3:**
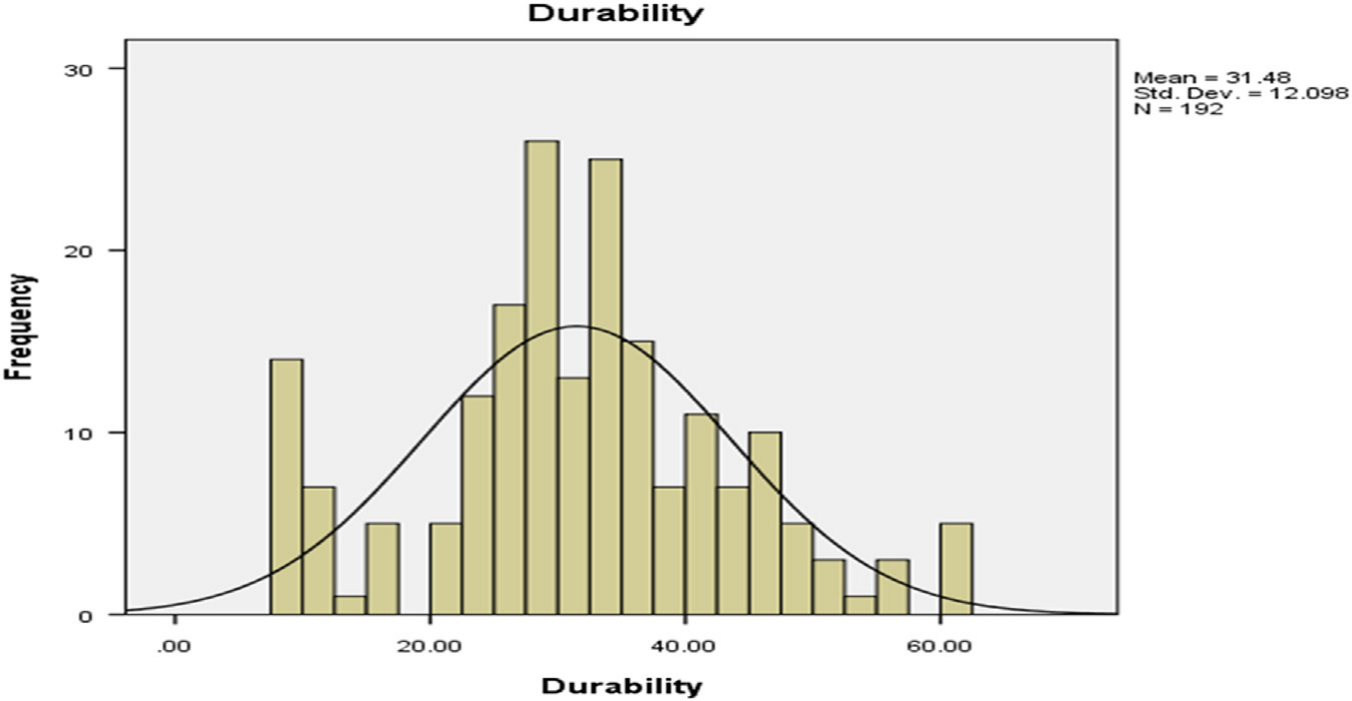
the durability of cassava pellets.

**Fig. 4:**
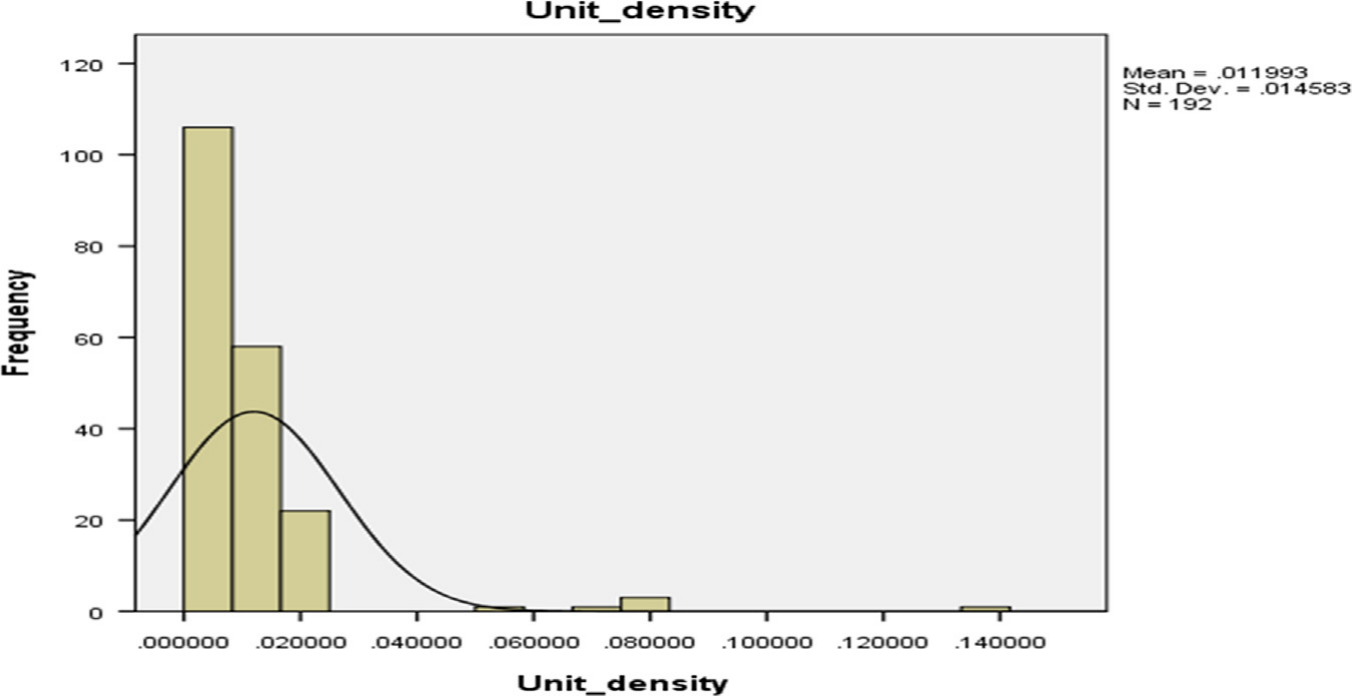
The unit density of cassava pellets.

Histograms for the hardness, bulk density, durability and unit density of cassava pellets are presented in Fig. 1, Fig. 2, Fig. 3, Fig. 4 respectively.

The parameters on Fig. 1, Fig. 2, Fig. 3, Fig. 4 are contained in Table 1 and normal plots on the figures showed how the distributions were fitted by the normal distribution. Other distributions may be applied when the raw data is analyzed further.

## 2 Experimental design, materials and methods

Several studies have been conducted on the pellets [1], [2], [3], [4], [5], [6], [7], [8], [9]. Similar data articles on pellets that applied statistical tools can be helpful, readers are referred to [10], [11], [12], [13], [14], [15], [16], [17], [18].

The materials used for this experiment are classified into two groups namely: the cassava powder and the mechanical extrusion rig.

### 2.1 Cassava preparation

**Fig. 5:**
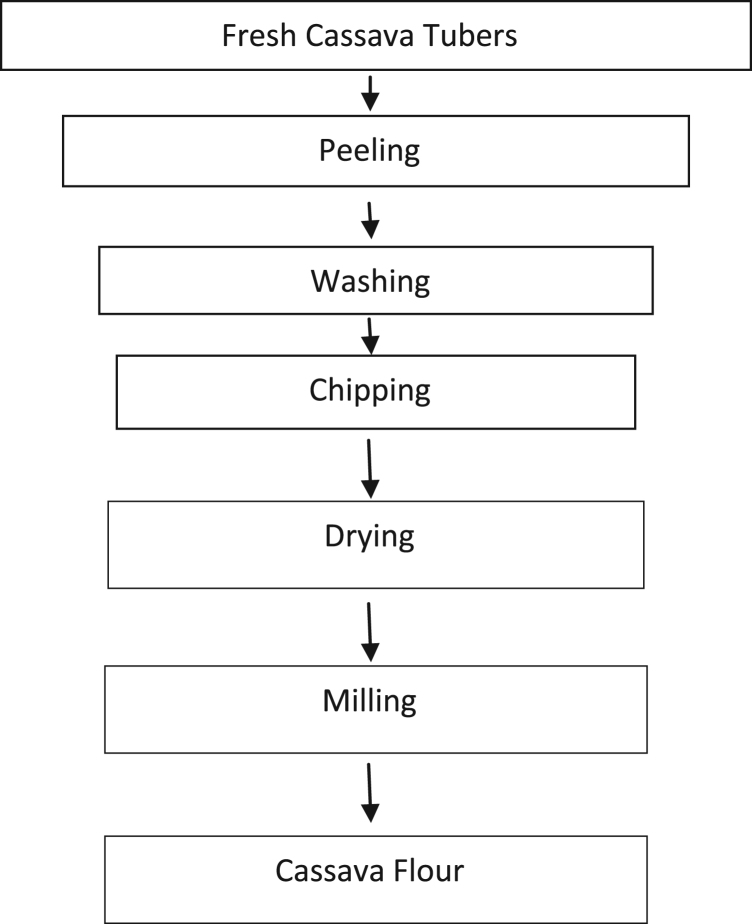
Flow chart for the processing of cassava tubers into cassava powder.

Cassava tubers were bought from Idofian market in Ifelodun Local Government area of Kwara State Nigeria. The tubers were processed into cassava powder as shown in Fig. 5. The moisture content of the cassava powder was 10%wb and it was conditioned to form cassava dough using Eq. (1). Weight of water ($W_{w}$) to be added is

(1) $$W_{w}=\left[\frac{100\;-\;M_{p}}{100\;-\;M_{g}}\;-\;1\right]\times W_{g}$$

where

$M_{p}=$Present moisture content
$M_{g}=$Required moisture content
$W_{g}=$Weight of sample in grams

### 2.2 Pelletization process

**Fig. 6:**
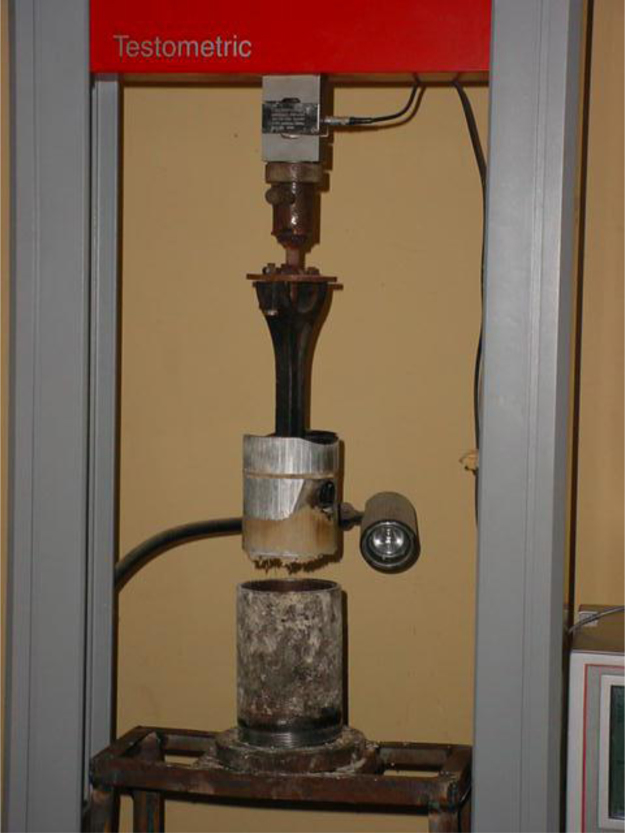
Showing the Piston-Cylinder Assembly on the Universal Testing Machine.

**Fig. 7:**
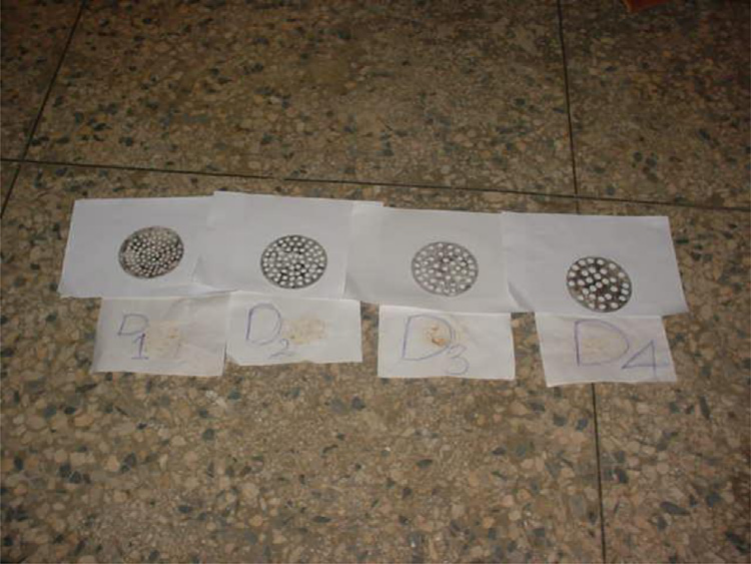
Showing the dies.

The mechanical extrusion process (pelletization) involves the application of a compressive force on the cassava dough enclosed in a cylinder with replaceable die called pelletization rig and is shown in Fig. 7. The pelletization rig containing the cassava dough was mounted on the “TESTOMETRICS” universal testing machine (model M500 50kN) as shown in Fig. 6 and extraction process took place on the “TESTOMETRICS” Universal testing machine.

#### 2.2.1 Description of the TESTOMETRICS universal testing machine

As shown in Fig. 6, the mechanical extrusion rig consists of two parts. The TESTOMETRICS universal testing machine (UTM Model M500 50KN, England, United Kingdom) and a piston cylinder rig which has been in use for extrusion purposes on the UTM. The UTM consists of the control console, load frame. crosshead, load cell, a computer and printer.

The load frame of the U T M is an extrusion support column with the slot for accessory mounting (cross head) twin re-circulatory ball screws. The cross-head range is 0.001 to 500 mm/min. Maximum cross head travels is 1000 mm. Load cells (load indicating mechanism) are automatically identified and have 800% overload protection capacity.

The machine can be programmed with 100 different test methods/definitions for quick menu recall using Win test software. Results, statistics and graphs can be generated with or without the use of a computer with optional long term data storage and retrieval. Test model/type includes tension, compression, flexural, cyclic etc with appropriate grip and fixtures available for each test type.

#### 2.2.2 The piston cylinder assembly

The piston cylinder assembly (shown in Fig. 6) is made up of three major components: the compression piston, the press cage cylinder, and the supporting platform.

The press cage cylinder is made of mild steel pipe with inside diameter of 160 mm, length of 105 mm, and thickness of 6 mm.

The compression piston is made up of mild steel of 104.23 mm diameter and 126.33 mm height. The supporting platform was made up of angle iron of 3 mm thick, inside dimension of 35 mm by 20 mm and 25 mm height. The dies were four in number; cut from 4 mm thick mild steel plate with holes of 6 mm diameter, 8 mm diameter, 10 mm diameter and 12 mm diameter, with circumference forming about 8 percent of the total area of the plate to cover the cylinder with wire quase, which was improvised for the collection of pellets formed for carefulness and ease of drying in the batch drier.

### 2.3 Data analysis

The 4^3^ factorial experiment design with randomized complete block design was adopted for the analysis. 4^**3**^ factorial design implies three (3) factors (moisture content, speed and die diameter) each at four (4) levels. The factor and levels are: moisture content (48.5%(wb), 50.5%(wb), 52.5%(wb), 54.5%(wb)), machine speed (1.5 mm/min, 2.5 mm/min, 3.5 mm/min, 4.5 mm/min), die diameter of the rig (6 mm, 8 mm, 10 mm, 12 mm). Each of these experiments was replicated three times. The total units of experiment were 4×4×4×3 which is 192 altogether. Analysis of variance (ANOVA) table was derived on each output (hardness, bulk density, durability and unit density).

Four moisture contents of 48.5%wb, 50.5%wb, 52.5%wb and 54.5%wb were therefore obtained altogether with 10%wb corresponding to the initial moisture.

Table 2, Table 6, Table 10 present the analysis of variance results for hardness, bulk density and durability of cassava pellets respectively. It was observed from the three tables that all their main factor effects as well as two and three ways interactions are significant at 5% level. This means that the hardness, bulk density and durability of cassava pellets respectively depend on the moisture content of the cassava mash, the machine speed, the die diameter of the rig and the combinations of these factors in pairs as well as three altogether.

**Table 2:** Analysis of variance for hardness of cassava pellets.

Tests of Between-Subjects Effects					
Source	Type III Sum of Squares	df	Mean Square	F	Sig.
Corrected Model	7561.947^a^	63	120.031	14.375	0.00
Intercept	23023.470	1	23023.470	2757.216	0.00
Moisture content	3068.848	3	1022.949	122.505	0.00
Speed	293.327	3	97.776	11.709	0.00
Die diameter	255.827	3	85.276	10.212	0.00
Moisture content * Speed	316.960	9	35.218	4.218	0.00
Moisture content * Die diameter	950.293	9	105.588	12.645	0.00
Speed * Die diameter	477.897	9	53.100	6.359	0.00
Moisture content * Speed * Die diameter	2198.796	27	81.437	9.753	0.00
Error	1068.833	128	8.350		
Total	31654.250	192			
Corrected Total	8630.780	191			

a R Squared = .876 (Adjusted R Squared = .815), Dependent Variable: Hardness.

**Table 3:** Post hoc test for significant differences in moisture content under hardness of cassava pellets in %(wb).

Waller-Duncan				
Moisture content	N	Subset		
		1	2	3
54.5%(wb)	48	4.9479		
52.5%(wb)	48		9.7917	
48.5%(wb)	48			13.8542
50.5%(wb)	48			15.2083

**Table 4:** Post hoc test for significant differences in machine speed under hardness of cassava pellets in mm|min.

Waller-Duncan				
Speed	N	Subset		
		1	2	3
1.5 mm|min	48	9.2708		
2.5 mm|min	48		10.8333	
3.5 mm|min	48		10.9375	
4.5 mm|min	48			12.7604

**Table 5:** Post hoc test for significant differences in die diameter under hardness of cassava pellets in mm.

Waller-Duncan			
Die_diameter	N	Subset	
		1	2
10 mm	48	9.7917	
12 mm	48	9.8438	
8 mm	48		11.7708
6 mm	48		12.3958

**Table 6:** Analysis of variance for bulk density of cassava pellets.

**Tests of Between-Subjects Effects**					
Dependent Variable: Bulk_density					
Source	Type III Sum of Squares	df	Mean Square	F	Sig.
Corrected Model	6697.337^a^	63	106.307	5.773	0.000
Intercept	622198.220	1	622198.220	33787.909	0.000
Moisture content	1458.475	3	486.158	26.400	0.000
Speed	422.526	3	140.842	7.648	0.000
Die diameter	1413.111	3	471.037	25.579	0.000
Moisture content * Speed	923.230	9	102.581	5.571	0.000
Moisture content * Die diameter	756.710	9	84.079	4.566	0.000
Speed * Die diameter	550.249	9	61.139	3.320	0.001
Moisture content * Speed * Die diameter	1173.036	27	43.446	2.359	0.001
Error	2357.097	128	18.415		
Total	631252.654	192			
Corrected Total	9054.434	191			

a R Squared = .740 (Adjusted R Squared = .612).

**Table 7:** Post hoc test for significant differences in moisture content under bulk density of cassava pellets in %(wb).

Waller-Duncan			
Moisture content	N	Subset	
		1	2
48.5%(wb)	48	54.1785	
50.5%(wb)	48	55.6527	
52.5%(wb)	48	56.3754	
54.5%(wb)	48		61.4990

**Table 8:** Post hoc test for significant differences in machine speed under bulk density of cassava pellets in mm|min.

Waller-Duncan			
Speed	N	Subset	
		1	2
4.5 mm|min	48	54.5113	
1.5 mm|min	48		57.0598
2.5 mm|min	48		57.6498
3.5 mm|min	48		58.4848

**Table 9:** Post hoc test for significant differences in die diameter under bulk density of cassava pellets in mm.

Waller-Duncan			
Die_diameter	N	Subset	
		1	2
12 mm	48	54.0167	
10 mm	48	54.4640	
6 mm	48		59.1229
8 mm	48		60.1021

**Table 10:** Analysis of variance for durability of cassava pellets.

**Tests of Between-Subjects Effects**					
Source	Type III Sum of Squares	df	Mean Square	F	Sig.
Corrected Model	26758.625^a^	63	424.740	45.452	0.00
Intercept	190318.009	1	190318.009	20366.381	0.00
Moisture content	16320.740	3	5440.247	582.174	0.00
Speed	1296.492	3	432.164	46.247	0.00
Die diameter	3716.795	3	1238.932	132.581	0.00
Moisture content * Speed	1842.024	9	204.669	21.902	0.00
Moisture content * Die diameter	1335.656	9	148.406	15.881	0.00
Speed * Die diameter	407.097	9	45.233	4.840	0.00
Moisture content * Speed * Die diameter	1839.820	27	68.141	7.292	0.00
Error	1196.123	128	9.345		
Total	218272.758	192			
Corrected Total	27954.748	191			

a R Squared = .957 (Adjusted R Squared = .936), Dependent Variable: Durability.

**Table 11:** Post hoc test for significant differences in moisture content under durability of cassava pellets in %(wb).

Waller-Duncan					
Moisture_content	N	Subset			
		1	2	3	4
54.5%(wb)	48	18.7585			
52.5%(wb)	48		28.7656		
50.5%(wb)	48			34.1346	
48.5%(wb)	48				44.2771

**Table 12:** Post hoc test for significant differences in machine speed under durability of cassava pellets in mm|min.

Waller-Duncan				
Speed	N	Subset		
		1	2	3
3.5 mm|min	48	27.6985		
2.5 mm|min	48		30.4727	
4.5 mm|min	48			33.6046
1.5 mm|min	48			34.1600

**Table 13:** Post hoc test for significant differences in die diameter under durability of cassava pellets in mm.

Waller-Duncan					
Die_diameter	N	Subset			
		1	2	3	4
12 mm	48	26.2444			
10 mm	48		28.1465		
8 mm	48			34.7581	
6 mm	48				36.7869

Table 14 presents the analysis of variance result for the unit density of cassava pellets. However, only the die diameter and the three-way interaction are significant at 5% level. This implies that the unit densities of cassava pellets only depends on the die diameter of the rig and the effect of the combination of moisture content, machine speed and die diameter of the rig.

**Table 14:** Analysis of variance for unit density of cassava pellets.

**Tests of Between-Subjects Effects**					
Source	Type III Sum of Squares	Df	Mean Square	F	Sig.
Corrected Model	0.020^a^	63	0.000	1.960	0.001
Intercept	0.028	1	0.028	170.986	0.000
Moisture content	0.001	3	0.000	1.327	0.269
Speed	0.000	3	0.000	0.879	0.454
Die diameter	0.007	3	0.002	15.371	0.000
Moisture content * Speed	0.002	9	0.000	1.227	0.284
Moisture content * Die diameter	0.001	9	0.000	0.890	0.537
Speed * Die diameter	0.001	9	0.000	0.821	0.598
Moisture content * Speed * Die diameter	0.007	27	0.000	1.641	0.036
Error	0.021	128	0.000		
Total	0.068	192			
Corrected Total	0.041	191			

a R Squared = .491 (Adjusted R Squared = .240), Dependent Variable: Unit density.

Table 3, Table 4, Table 5, Table 7, Table 8, Table 9, Table 11, Table 12, Table 13 present post hoc test for significant differences in the levels of moisture content, machine speed and die diameter of the rig for hardness, bulk density and durability of cassava pellets respectively. Durable cassava pellets which can withstand stress during handling can be obtained at moisture content level above 48.5%wb and below 55.5%wb.

**Fig. 8:**
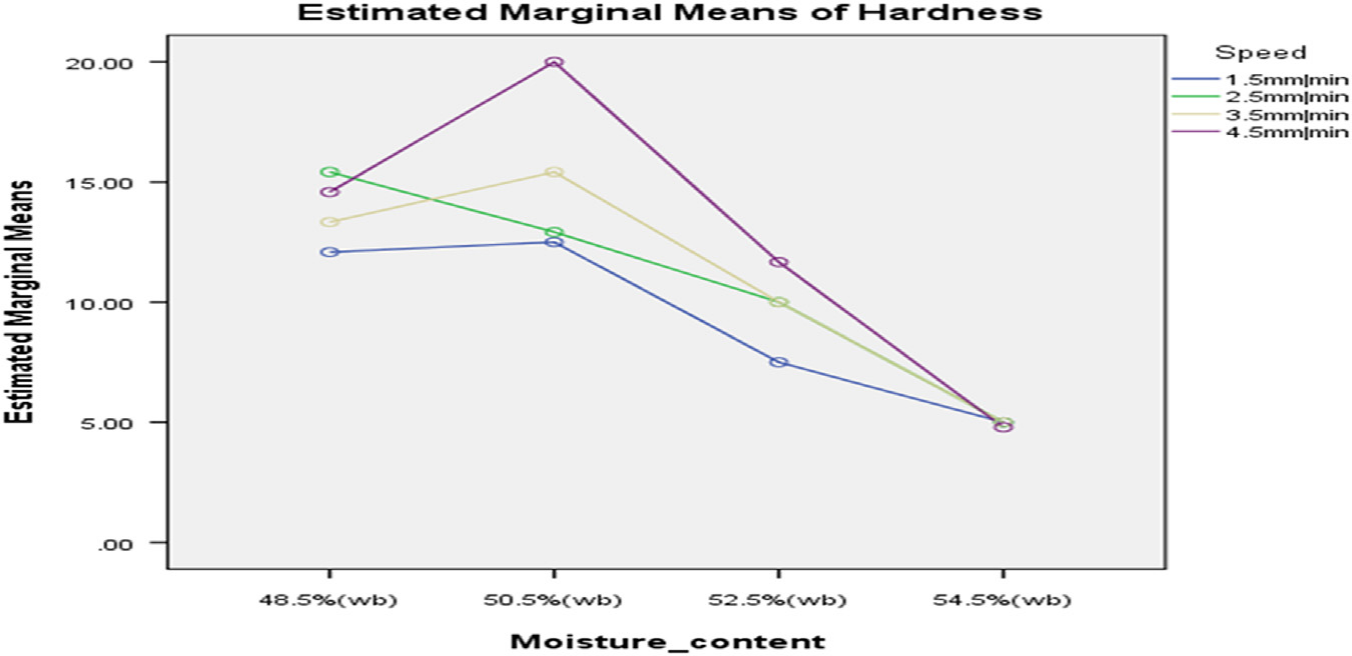
Graph of interactions between moisture content and machine speed on hardness of cassava pellets.

**Fig. 9:**
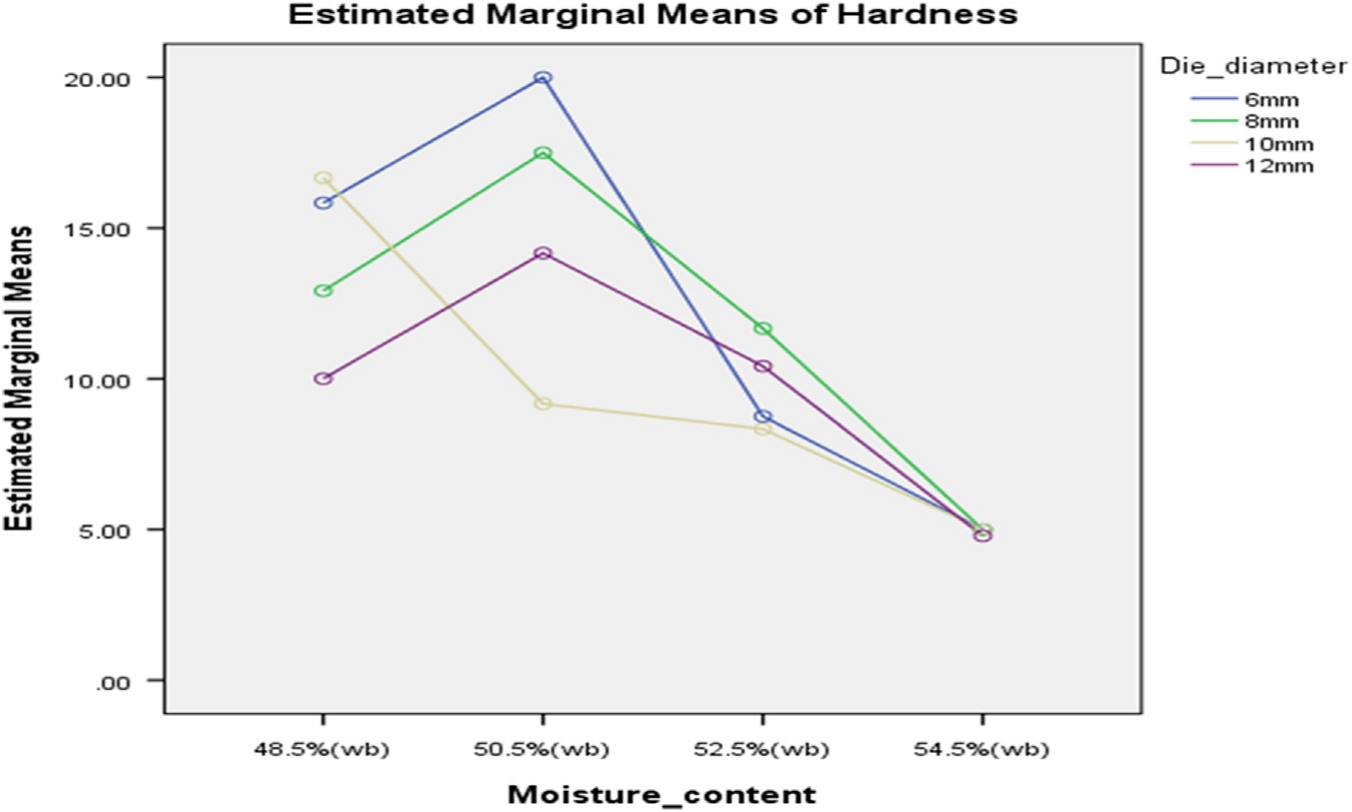
Graph of interactions between moisture content and die diameter on hardness of cassava pellets.

**Fig. 10:**
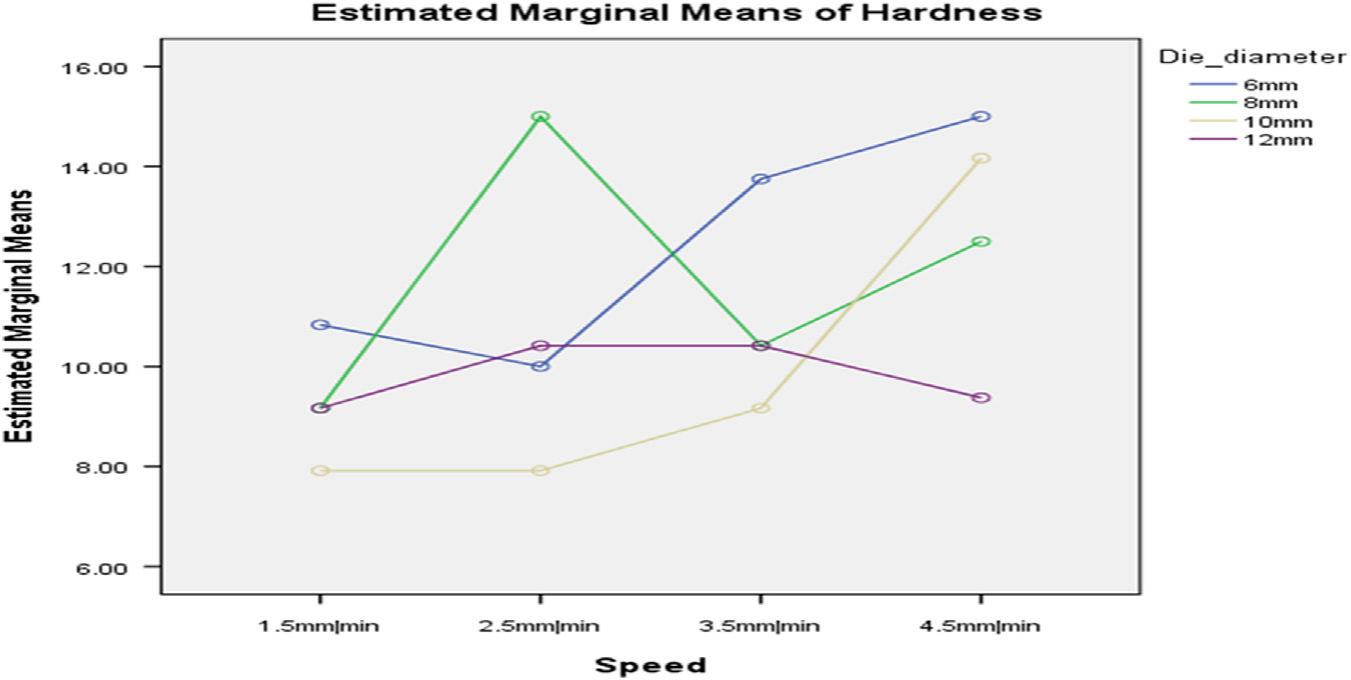
Graph of interactions between speed and die diameter on hardness of cassava pellets.

**Fig. 11:**
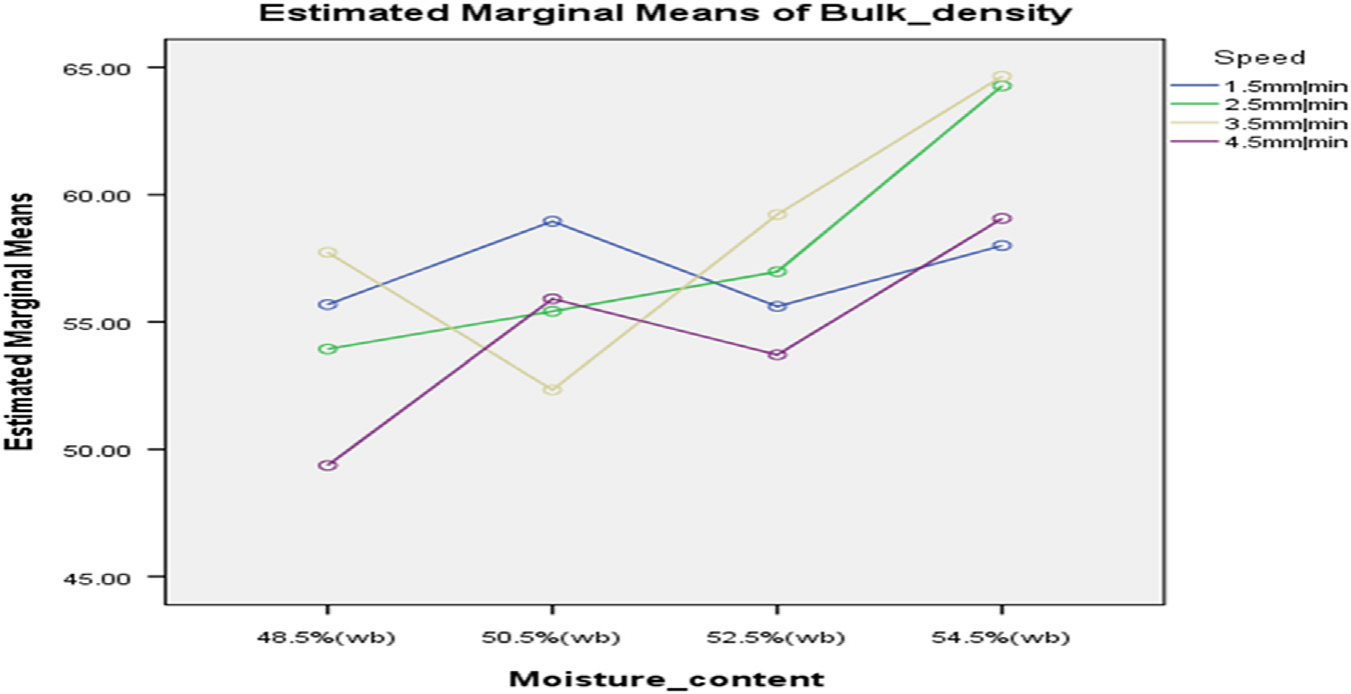
Graph of interactions between moisture content and machine speed on Bulk density of cassava pellets.

**Fig. 12:**
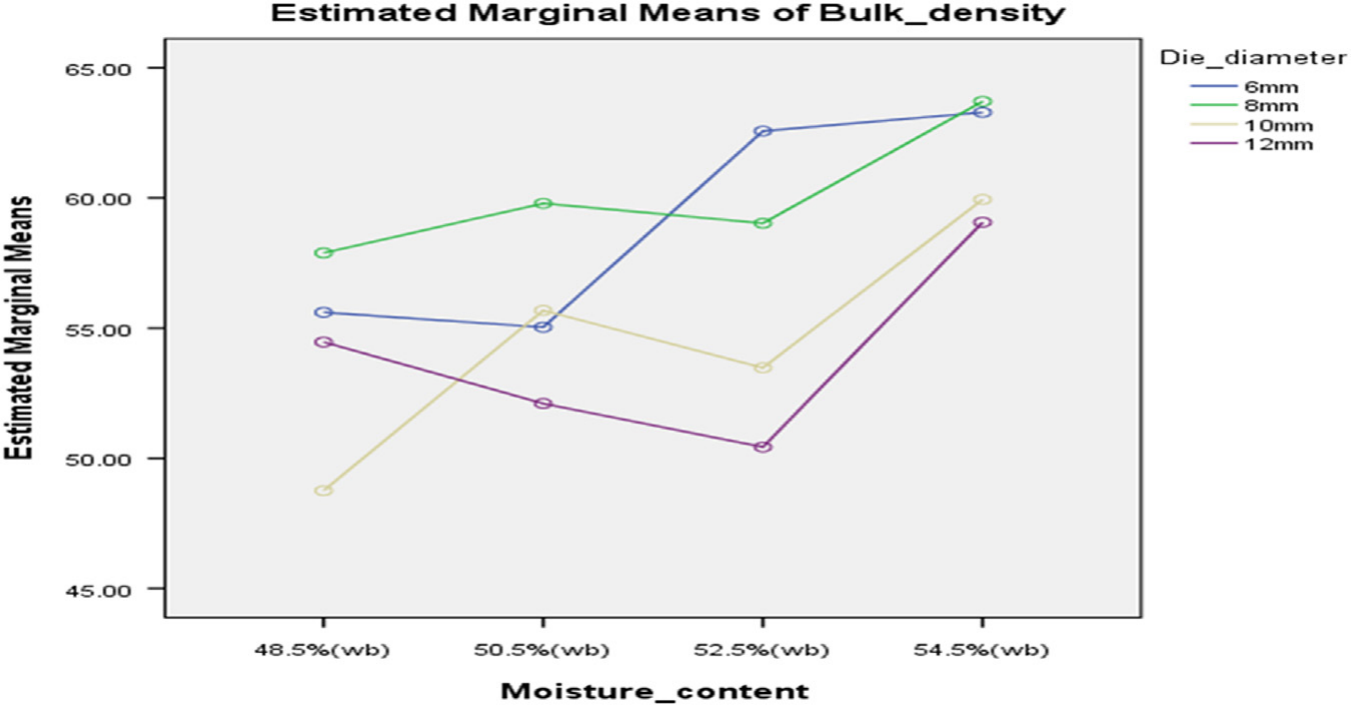
Graph of interactions between moisture content and die diameter on Bulk Density of cassava pellets.

**Fig. 13:**
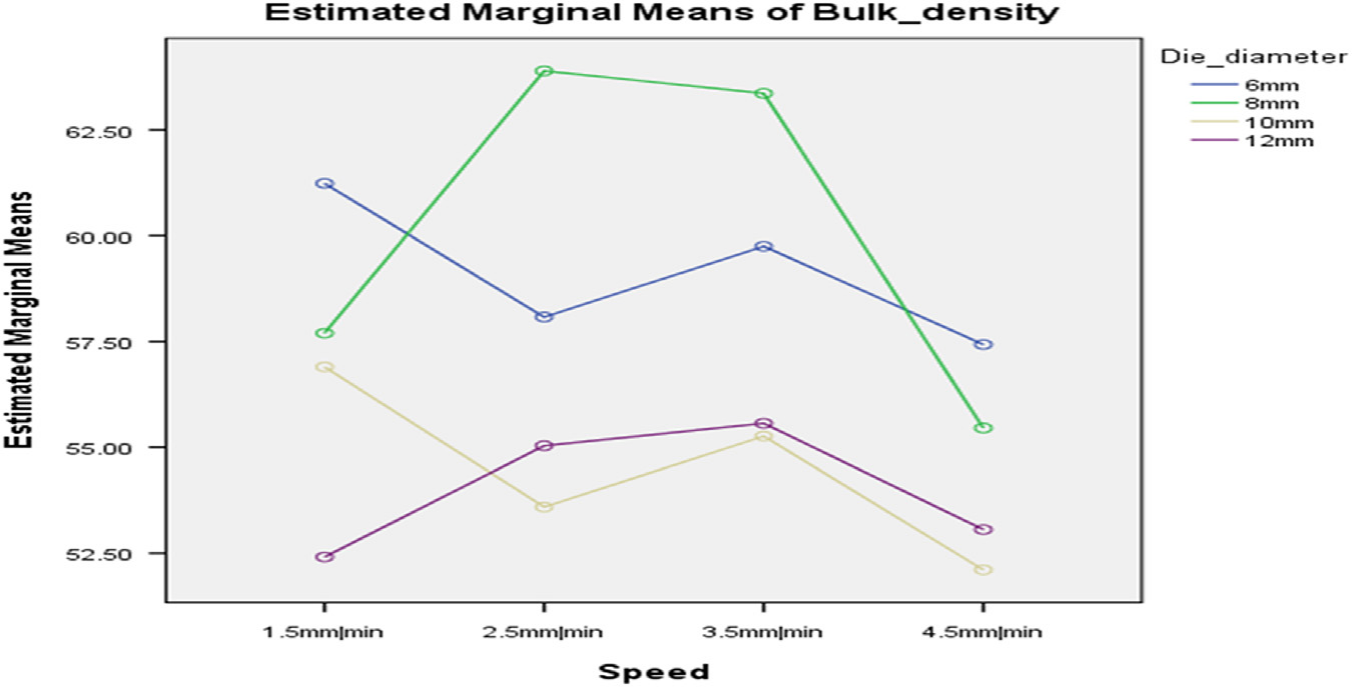
Graph of interactions between speed and die diameter on Bulk Density of cassava pellets.

**Fig. 14:**
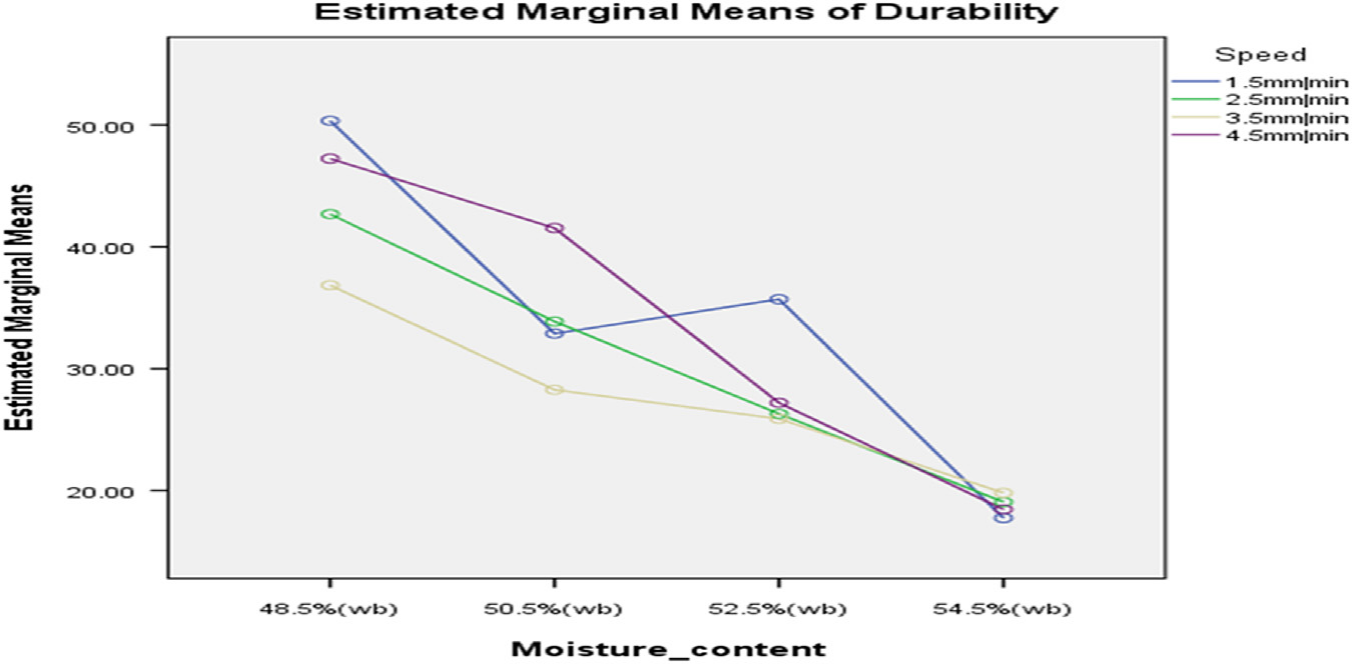
Graph of interactions between moisture content and machine speed on Durability of cassava pellets.

**Fig. 15:**
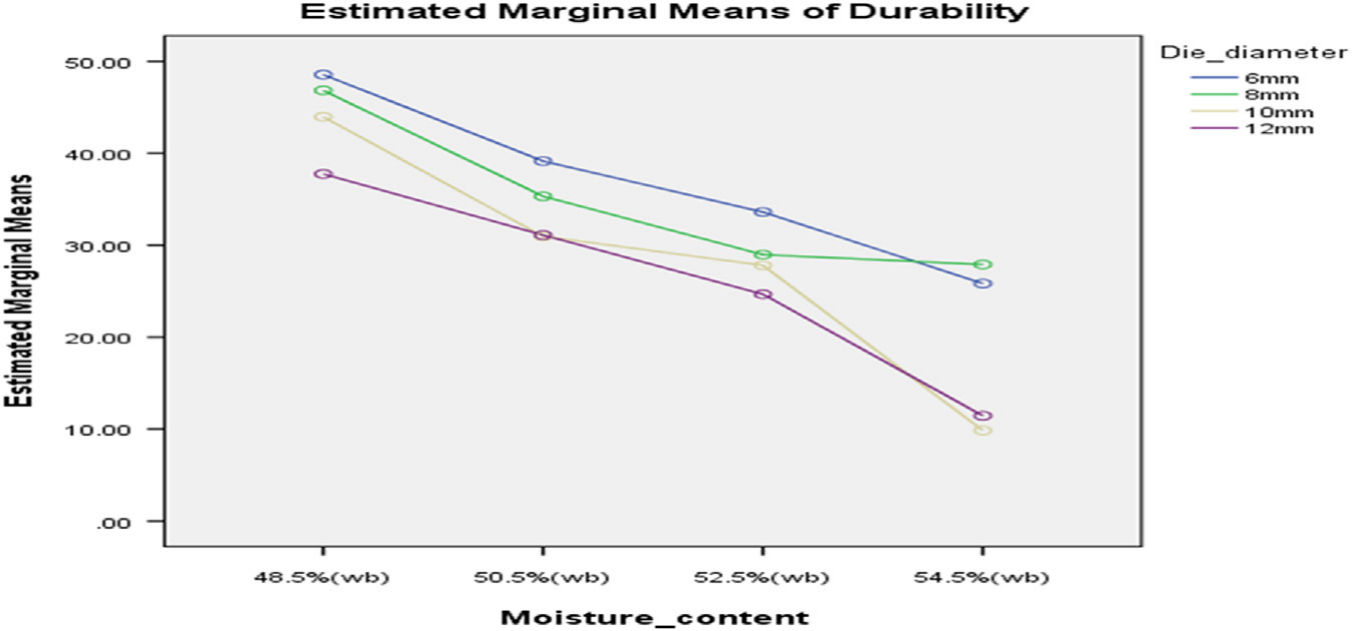
Graph of interactions between moisture content and die diameter on durability of cassava pellets.

**Fig. 16:**
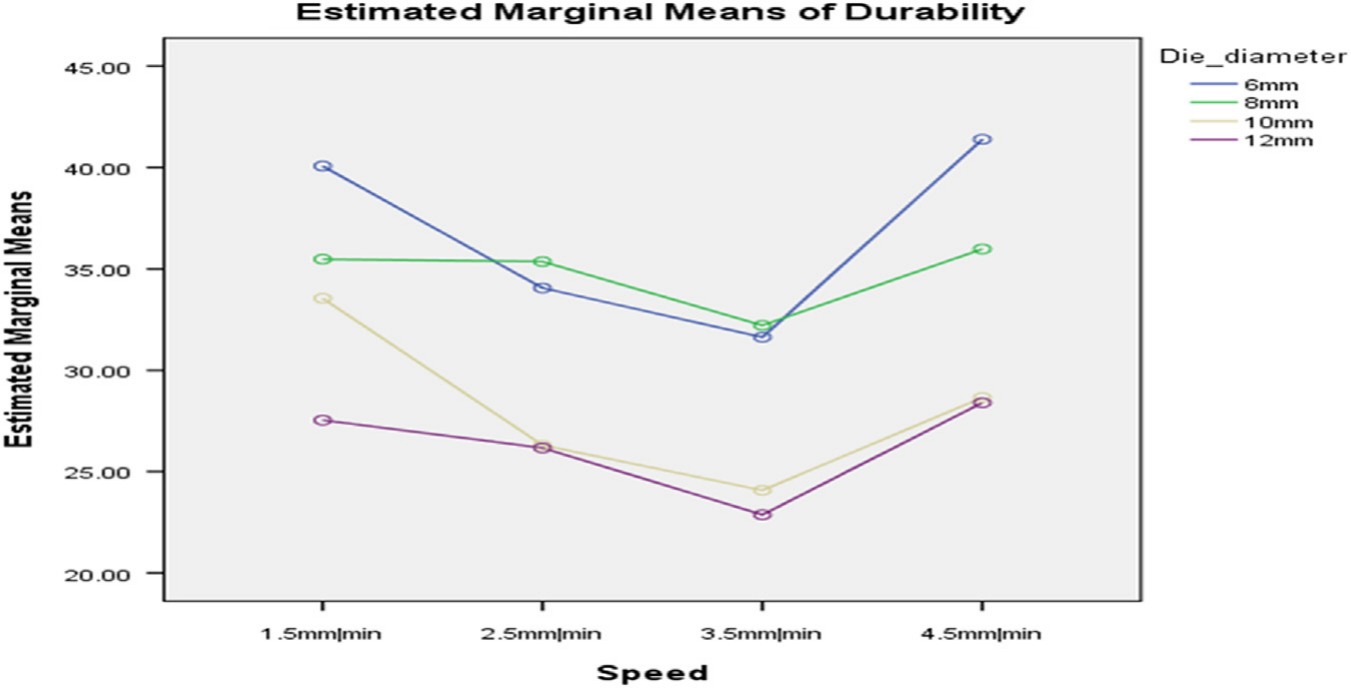
Graph of interactions between speed and die diameter on durability of cassava pellets.

Table 15 presents the post hoc test for significant differences in the levels of die diameter of the rig for unit density of cassava pellets. Likewise, Fig. 8, Fig. 9, Fig. 10, Fig. 11, Fig. 12, Fig. 13, Fig. 14, Fig. 15, Fig. 16 present the graphs for interactions between: moisture content and machine speed; moisture content and die diameter; machine speed and die diameter respectively on hardness, bulk density and durability of cassava pellets.

**Table 15:** Post hoc test for significant differences in die diameter under unit density of cassava pellets in mm.

Waller-Duncan			
Die diameter	N	Subset	
		1	2
8 mm	48	0.00689062	
12 mm	48	0.00864625	
10 mm	48	0.00980583	
6 mm	48		0.02263021

**Fig. 17:**
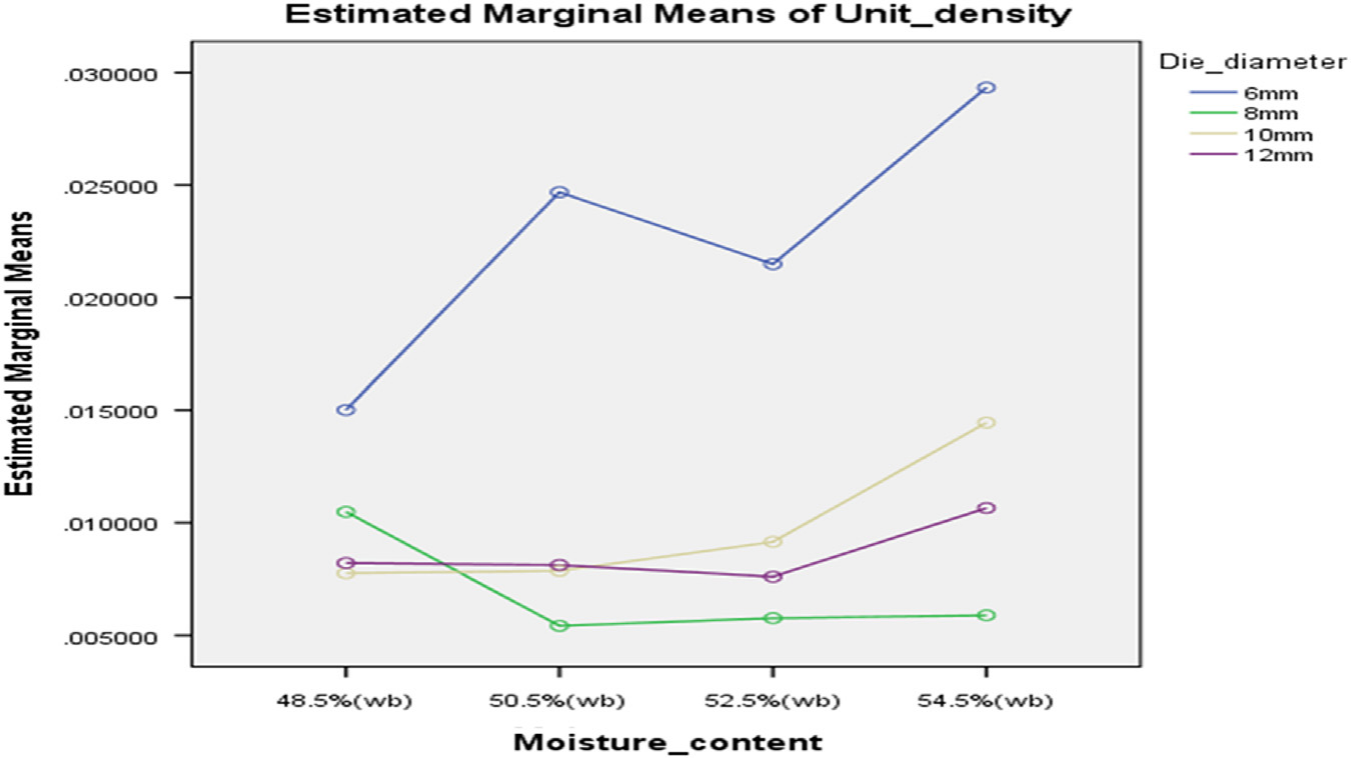
Graph of interactions between moisture content and die diameter on Unit Density of cassava pellets.

**Fig. 18:**
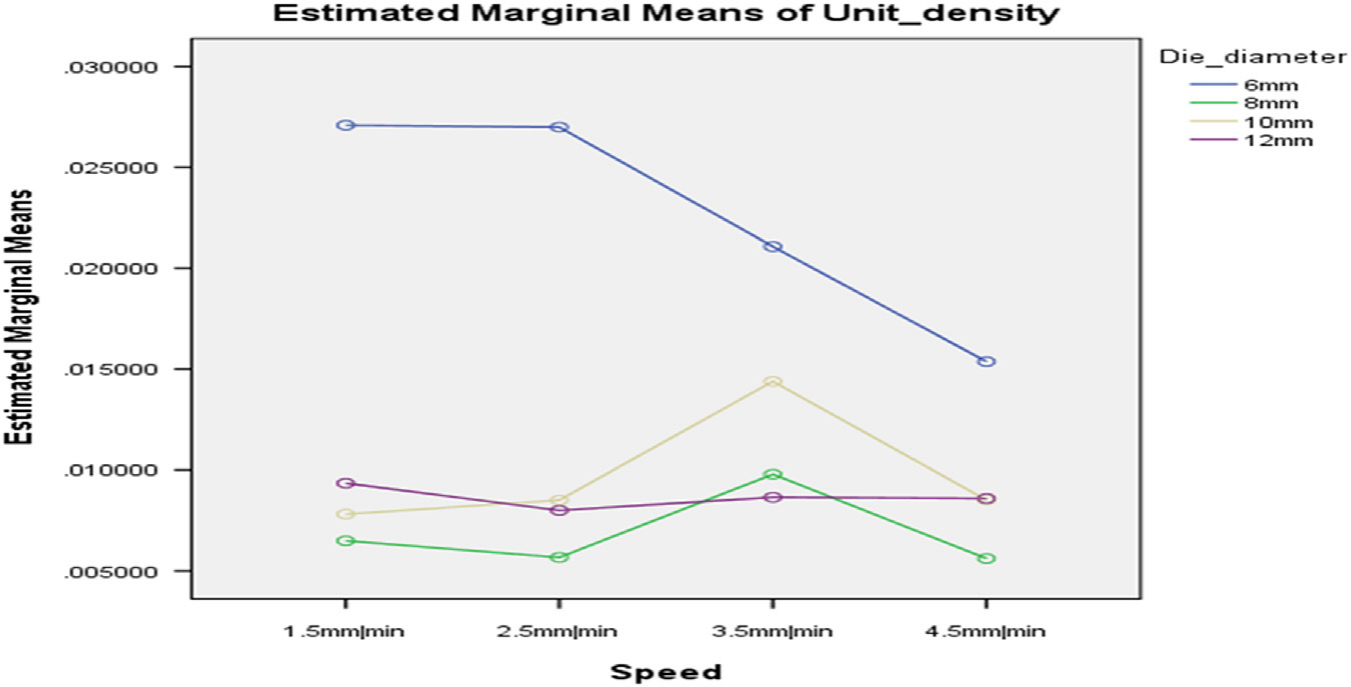
Graph of interactions between speed and die diameter on unit density of cassava pellets.

Lastly, Fig. 17, Fig. 18 present graphs for interactions between: moisture content and die diameter; machine speed and die diameter on unit density respectively.
